# Enhancing team process improvement through the implementation of the Functional Resonance Analysis Method (FRAM): the case of medication reconciliation at hospital discharge

**DOI:** 10.1186/s12913-026-15187-6

**Published:** 2026-07-29

**Authors:** Liselotte van Dijk, Linda van Eikenhorst, Cordula Wagner

**Affiliations:** 1 Netherlands Institute for Health Services Research (Nivel), PO Box 1568, Utrecht, 3500 BN Netherlands; 2 Amsterdam UMC, Vrije Universiteit Amsterdam, Department of Public and Occupational Health, Amsterdam Public Health Research Institute, De Boelelaan, Amsterdam, 1117 Netherlands

**Keywords:** Medication reconciliation, Team process improvement, Functional Resonance Analysis Method, Resilience

## Abstract

**Background:**

Healthcare professionals working in complex dynamic work systems are often forced to adjust their behaviour to changing conditions, possibly causing practice variation. Teams can analyse the (un)desirability of those variations by conducting team process improvement. This refers to a learning process in which teams reflect on past performances and formulate improvement strategies. The objective of this study was to identify barriers and facilitators for team process improvement when applying the Functional Resonance Analysis Method (FRAM).

**Methods:**

This study entails a multicentre prospective study. Nine cardiology and orthopaedic wards from eight Dutch hospitals participated in this study. For each ward, team process improvement consisted of four phases: 1) Constructing FRAM, 2) Discussing differences between protocols and daily practice, 3) Reflecting on intended improvements, 4) Evaluating team reflection process.

**Results:**

On all wards, we identified several discrepancies between protocol and daily practice. After sharing and discussing these results, most wards proposed improvements aimed at making the process safer and more efficient. Facilitators for team process improvement included: team composition (dedicated task forces and multidisciplinary teams) and being familiar with the analysed process. Identified barriers included a lacking motivation and limited involvement from the ward’s contact person in the study.

**Conclusions:**

This study showed that FRAM can provide valuable input to initiate team process improvement. The reflection process helped healthcare professionals to decide whether adjustments are needed to keep patients safe or if they introduced risks that were meant to be reduced by implementing protocols. To undertake successful team reflection, we suggest assembling dedicated task forces that regularly evaluate the process. The task force should consist of healthcare professionals from different disciplines.

## Introduction

The last decade, there has been growing interest in improving patient safety globally. Within the traditional idea of safety (Safety-I), safety is viewed as a state where nothing goes wrong or where the likelihood that something can go wrong is acceptably small [1]. This approach relies on following evidence-based guidelines and protocols to ensure quality and safety [2]. This view does not acknowledge that human performance often goes right because people constantly adjust their behaviour to changing conditions [3].

Recognizing this, there has been a shift towards a new perspective on patient safety known as Safety-II. This perspective understands daily practice as part of a complex adaptive system and focuses on the system’s ability to succeed under varying conditions [3, 4]. Within this framework, practice variation is seen as a logical consequence of the resilient behaviour that healthcare professionals must exhibit and is not necessarily undesirable [3–5]. Safety can be further improved by analysing the nature and effects of these variations.

A useful tool for analysing safety in complex systems from a Safety-II perspective is the Functional Resonance Analysis Method (FRAM) [6]. FRAM can be applied to identify differences between protocol and practice, determine when professionals are forced to adapt to everyday conditions, identify performance variability and make suggestions to (re-)design clinical processes and procedures [6, 7].

One healthcare process prone to variation in daily practice is medication reconciliation (MR) at discharge. MR entails compiling the most accurate list of a patient’s medication and to check this for unintended discrepancies, reconciling and documenting changes [8, 9]. MR reduces medication errors at admission and discharge, preventable adverse drug events (ADEs) 30 days post discharge and medication discrepancies [10, 11]. Earlier research revealed performance variability in several functions in the process of MR at discharge. To overcome barriers such as a lack of time or resources, healthcare professionals had to choose between working thoroughly or working swiftly [12]. These adjustments sometimes led them to, deliberately or accidentally, deviate from protocols. The question remains whether these changes and non-compliance to protocols keep patients safe or if they introduce potential risks.

Insights into this question can be provided by conducting team process improvement. Team process improvement refers to a learning process within teams by 1) reflecting on past team performances, and 2) formulating alternative strategies for improving those tasks [13, 14]. Performing MR at discharge is a process suitable for team process improvement because it requires collaboration between several healthcare professionals [12].

The objective of this study is to identify barriers and facilitators for team process improvement when applying FRAM.

## Methods

### Design

**Fig. 1:**
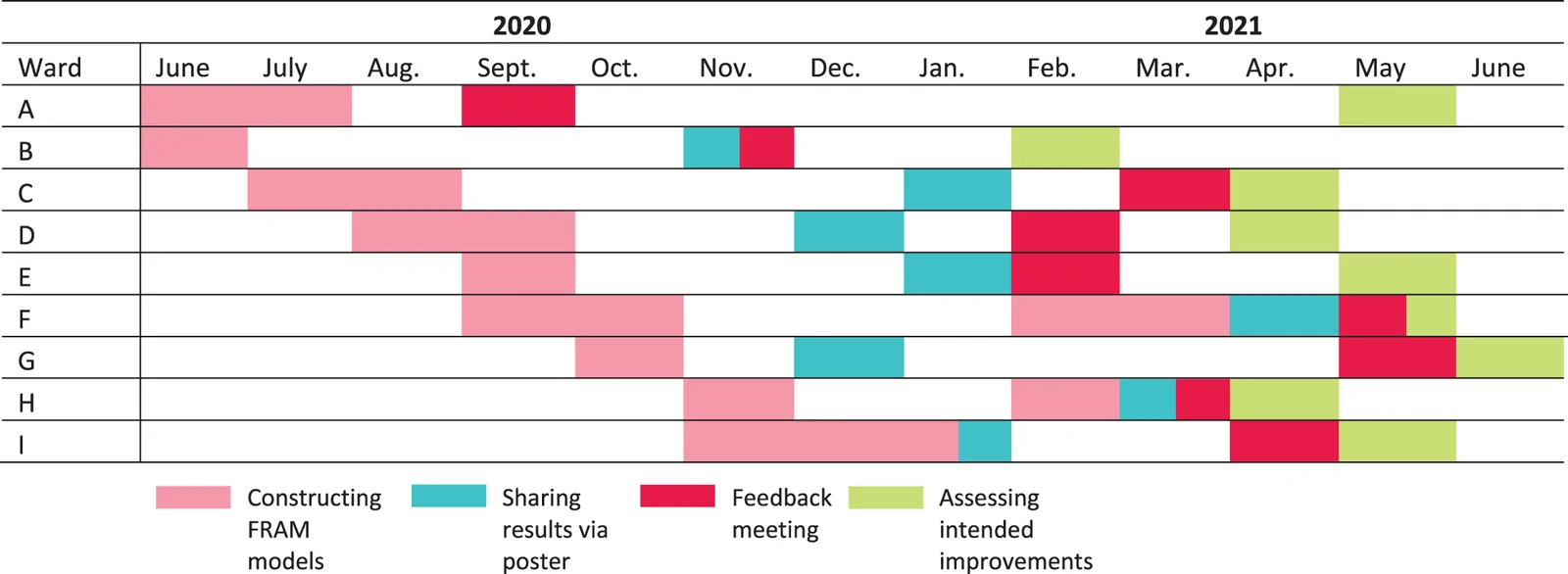
Timeline for each ward

This study is registered at Netherlands Trial Register (registration number: NL8778), and the published study protocol describes the study methods in detail [15]. This protocol was drafted before the COVID-19 pandemic emerged. Therefore, we had to adjust our study design in some ways (see Box 1). Data collection took place over 17 months to enable inclusion of multiple wards (see Fig. 1). Hospitals were invited to participate in the three studies described in the study protocol. Every two months, one or two wards enrolled in the study. In this study, we focused on a discharge process. Therefore, we wanted to enrol wards in which patients’ hospital stay was relatively short. We decided cardiology and orthopaedic wards were eligible for participation, because of high turnover of patients. Patients or the public were not involved in the design, or conduct, or reporting, or dissemination plans of our research.

**Box 1:** Adjustments due to the COVID-19 pandemic

• The randomized stepped wedge design could not be applied. Enrolment in the study was based on the ward’s availability instead of following the order of earlier randomization, and the study period was extended. Therefore, we did not determine the effect of team process improvement on safety indicator outcomes.	
• The FRAM interviews and feedback meetings mostly took place digitally instead of on-site.	
• Most wards were unable to organize a feedback meeting shortly after the FRAM models were drafted. Therefore, we first send them a poster with the main findings. Wards unable to find the time for an interactive meeting, received a pre-recorded presentation explaining the results.	
• Our initial plan was to measure employee resilience before and after the FRAM intervention. This was not feasible due to the increased workload and scattered data collection on the participating wards, which led to a less clear cut-off point of the intervention period.	

### Data collection

For each ward, team process improvement consisted of four phases:

1. Constructing FRAM models.
2. Discussing differences between protocols and daily practice.
3. Reflecting on intended improvements.
4. Evaluating team reflection process.

#### Phase 1: Constructing FRAM models

First, we constructed FRAM models that describe how MR should be performed at discharge. These Work-As-Imagined/WAI models were based on national Dutch guidelines and hospital protocols provided by each contact person. Second, we constructed a Work-As-Done/WAD model for each ward, to show how MR at discharge is performed in daily practice. Input for these models was collected through interviews with all healthcare professionals involved (physicians, nurses and pharmacy technicians). They were asked to describe the process in detail, focusing on their role and the adaptations needed. FRAM interviews were audio recorded and subsequently transcribed. All interviewees received an interview summary and were asked to check whether their input was interpreted correctly. A more detailed description of the construction process can be found in our other publication [12].

#### Phase 2: Discussing differences between protocols and daily practice

The authors compared the WAI and WAD model for each ward and presented this analysis during a feedback meeting with HCPs from the participating wards, pharmacy staff, and quality advisors. During this meeting, the teams could provide feedback on the accuracy of the models presented. We discussed the barriers for performing MR at discharge and participants were asked to reflect on the (un)desirability of the discrepancies identified. The feedback meeting was audio recorded and the first author made field notes.

#### Phase 3: Reflecting on intended improvements

Following the feedback meeting, we asked the teams to further reflect on the results and to, if they deemed it necessary, formulate suggestions for improvements. Wards were asked to report their suggestions in an action plan. Topics in this template were: the recognisability of the Work-as-Done model, (un)desirability of the discrepancies identified and intended changes.

#### Phase 4: Evaluating team reflection process

Each ward provided a contact person we could turn to for questions and who was involved in the entire research process. We conducted an interview with this contact person to evaluate how they had experienced the research period. An interview guideline was used. Questions were aimed at whether the FRAM models had provided insights into discrepancies between protocols and practice and if their team was motivated to reflect on these discrepancies. All interviews were audio recorded and subsequently transcribed.

#### Ward characteristics and employee resilience

We collected additional measurements to better understand the context of each participating hospital. Contact persons reported several hospital and ward characteristics in a Microsoft Excel file. We also collected characteristics among nurses working at the participating wards. Nursing experience (in years) was registered and we measured employee resilience using the most recent version of the Employee Resilience Scale (EmpRes) [16]. The online questionnaire consisted of eighteen statements about individual and team resilience which had to be rated using a 5-point Likert scale (1 = low resilience, 5 = high resilience) [17]. It is relevant to collect these measurements as nurses with high levels of resilience may be more likely to adjust their work to match changing conditions, causing them to deviate from protocols.

### Data analysis

We created one Microsoft Excel file that included the main results of each phase. For each ward, we noted whether differences between protocols and practice were extensive (phase 1), how they reflected on the discrepancies shared during the feedback meeting (phase 2), which improvements they proposed or implemented (phase 3), and how they evaluated the team reflection process (phase 4).

#### Phase 1: Constructing FRAM models

We constructed FRAM models to visualize all activities within the process of MR at discharge and their interdependencies. After studying the ward protocols and rereading the interview transcripts, two researchers translated all the different steps in the process into functions and, if possible, added the relevant FRAM aspects: Input, Output, Precondition, Resources, Time, and Control. The collected documents were used to construct 17 FRAM models in total using FRAM Model Visualiser Pro [18]. One general WAI model was constructed based on two national guidelines for MR and the transfer of medication information from secondary to primary care [19, 20]. For all eight wards, a separate WAI and WAD model was constructed. We then compared both models per ward to identify any possible discrepancies. By comparing the Work-as-Imagined and Work-as-Done models, we hoped to gain insight into why and when healthcare professionals (un)deliberately deviate from protocols.

#### Phase 2: Discussing differences between protocols and daily practice

The highlights of each audio recording of the feedback meeting and field notes were noted for each ward.

#### Phase 3: Reflecting on intended improvements

We collected all proposed and implemented improvements and made an overview for each ward.

#### Phase 4: Evaluating team reflection process

Transcripts from evaluation interviews were analysed in MAXQDA version 11. After familiarisation with the data, a coding scheme was developed by the researchers and the transcripts were coded following the principles of thematic analysis as described by Braun & Clarke (2006). To test the coding scheme developed, three interviews were double coded. After the first author analysed the remaining interviews, the codes were clustered into overarching themes. These themes form the basis for reporting the findings for this phase. The themes were: identified barriers and facilitators for team process improvement, proposed and implemented improvements, accuracy of WAD models and (un)desirability of practice variation.

#### Ward characteristics and employee resilience

Ward characteristics were presented in a table to provide an overview per hospital. To analyse the data from the EmpRes questionnaire, we used descriptive statistics (means and standard deviations) to report individual and team resilience. Analyses were performed using Stata version 16.

### Ethics

The Medical Ethics Committee of the VU University Medical Centre Amsterdam declared that the study was not subjected to Medical Scientific Research with humans (WMO) and determined that the Medical Research Involving Human Subjects Act did not apply to this study (number 2019.571). The need for ethical approval was waived by the Medical Ethics Committee of the VU University Medical Centre Amsterdam. Informed consent was obtained as stated in the published study protocol [15]. A signed informed consent form was required from the hospital’s board of directors to enrol in the study. Prior to all interviews, verbal informed consent was obtained. Consent was collected verbally because all contact between the researcher and interviewees was conducted digitally or by phone. The Medical Ethics Committee of the VU University Medical Centre Amsterdam approved this procedure.

## Results

### Participating wards

Nine cardiology and orthopaedic wards from eight Dutch hospitals participated in this study (see Table 1). Hospitals varied in hospital type and geographical location. In all participating hospitals, a hospital pharmacy was present. The number of beds per ward varied between 15 and 50 and the number of discharges also varied widely [2–28].

**Table 1:** Characteristics of the participating hospitals and wards

	Hospital 1			Hospital 2	Hospital 3	Hospital 4	Hospital 5	Hospital 6	Hospital 7	Hospital 8
	Ward A	Ward G	Ward B		Ward C	Ward D	Ward E	Ward F	Ward H	Ward I
*Hospital information*										
Hospital type	Academic	Academic	Tertiary teaching		General	Tertiary teaching	General	Academic	Tertiary teaching	General
Hospital pharmacy	Yes	Yes	Yes		Yes	Yes	Yes	Yes	Yes	Yes
Outpatient pharmacy	Yes	Yes	Yes		No	Yes	Yes	Yes	Yes	No
*Ward information*										
Number of beds on the ward	34	32	36		50	35	23	49	31	15
Ward type	Cardiology	Orthopaedic	Cardiology		Orthopaedic	Orthopaedic	Cardiology	Orthopaedic	Cardiology	Orthopaedic
Mean number of discharges per day	2.5	2.5	6		26*	7	Unknown**	6	4.5	5

* This relatively high number of discharges can be explained because on this ward patients were often admitted for one day to undergo hip and knee replacements

** The contact person for this ward was unable to retrieve this information

The EmpRes questionnaire was sent to 372 nurses, 119 filled it out completely (response rate of 32%). Respondents worked on Cardiology (32.8%), Orthopaedics (23.5%) and Surgery (43.7%) wards. Overall, nurses scored individual (*M* = 3.8, SD = 0.7) and team (*M* = 3.6, SD = 0.7) resilience positively. Most respondents (35%) had between one and five years of work experience.

Figure 1 shows the study timeline for each ward. Wards gradually enrolled in the study. The consecutive activities were carried out based on availability of each ward, resulting in a scattered data collection. Due to COVID-19 related work pressure, the order of the activities in time varied.

### Phase 1: Constructing FRAM models

Between May 2020 and February 2021, 63 nurses, physicians and pharmacy technicians were interviewed about their role in the process of MR at discharge. Most interviewees were female (76%), and the median years of work experience was 6 years. A detailed description of the constructed models can be found in another publication [12]. When comparing WAI and WAD models, we noticed that WAD models consisted of more activities, had more involved healthcare professionals and the process was not as linear as described in WAI models. The results indicated that HCPs occasionally had to adjust their work in order to manage a lack of time or resources. In those instances, they had to decide between working thoroughly or working efficiently. Healthcare professionals made these assessments based on experience and knowledge.

### Phase 2: Discussing differences between protocols and daily practice

Because eight wards were unable to organise a feedback meeting within the designated time period, we decided to send them a poster in advance with the main findings. This included a WAD model, barriers and facilitators and action points. For seven wards, we then organized an (online) meeting with the nursing staff and, when possible, physicians, pharmacy staff and quality advisors. During the meeting, the first author presented both ward-specific FRAM models, including discrepancies between those models. Upon request, two wards received a pre-recorded presentation instead of a meeting. These wards were asked to discuss the results within their team.

### Phase 3: Reflecting on intended improvements

With the exception of one ward, all wards had proposed one or several improvements (Table 2). One ward had documented these changes in an action plan. The others did not have the time or motivation to do so. In most cases, the identified changes were derived from the barriers we found during this study and which we shared with the teams during the feedback meeting. Several changes were directed towards improving communication and collaboration between nurses, physicians and pharmacy technicians. On wards where nurses performed medication counselling, CPs wanted to explore the possibility of involving pharmacy technicians in this task. All changes were aimed at making the process safer and more efficient.

**Table 2:** Proposed or implemented improvements per ward

Ward number and type	Variations identified	Improvements proposed (P) or implemented (I)
A - Cardiology	Nurses do not always feel equipped to counsel patients about their discharge medications, which results in them consulting the pharmacy suddenly for complex discharges.	Pharmacy will be consulted for complex discharges (I).
	Some nurses would only focus on counselling the patient during discharge while others ensured family members were always present during discharge counselling.	All nurses will encourage family members to become more involved in the discharge procedure (P).
B - Cardiology	Variation in the way MR is performed for patients being discharged during weekends.	To establish one hospital wide procedure for patients being discharged during weekends (I).
C - Orthopaedic	Nurses do not always feel equipped to counsel patients about their discharge medications, which results in them consulting the pharmacy suddenly for complex discharges.	To explore the possibility for pharmacy technicians to perform patient counselling (P).
	When pharmacy technicians identify discrepancies during medication reconciliation, it takes a long time before they contact nurses to discuss the follow-up.	To improve communication between ward and pharmacy to reduce medication errors (P).
	The protocol is outdated, so work arrangements are communicated verbally and cannot be reviewed in written form.	A new protocol needs to be implemented (P).
D – Orthopaedic *(Action plan was filled out)*	Variation in whether physicians have initiated the discharge procedure when the patient is ready to be discharged.	Physicians will be reminded to initiate the discharge procedure at least 4 hours before discharge (P).
E - Cardiology	Not applicable	This ward did not propose or implement any improvements, because they said were satisfied with how they performed MR.
F - Orthopaedic	Some nurses did not know they could see whether an order had been placed for medication delivery. They would call the pharmacy to request this information, causing process variation.	Nurses will check patient files themselves to see when the pharmacy will deliver medications, instead of calling them multiple times a day (I).
G - Orthopaedic	A dedicated time slot was lacking when physicians would decide which patients could be discharged that day or the next day. This meant nurses and pharmacy technicians were unable to prepare for discharges, causing them to perform MR at the last minute.	To speed up the discharge process, physicians should focus on discharge planning during rounds (P).
H - Cardiology	MR is not always performed (correctly) at admission, which leads to additional tasks to be performed at discharge.	To ensure that MR at admission is performed correctly to avoid interactions (P).
	Nurses do not always feel equipped to counsel patients about their discharge medications, which results in them consulting the pharmacy suddenly for complex discharges.	To explore the possibility of involving the pharmacy in discharges (P).
I - Orthopaedic	Sometimes the medication overview has not been reviewed before discharge.	To remind physicians to check medications before a patient is discharged (P).

### Phase 4: Evaluating team reflection process

Because most CPs had not shared a filled-out action plan, proposed improvements were also discussed during this interview. Most contact persons (CPs) were pleased with FRAM as a research method to reflect on MR at discharge. They mentioned it was very important that all professions involved in this process were included in the study as MR is a multidisciplinary process. Using colours in the FRAM models to indicate who performed an activity emphasized the dependency between HCPs and the need for collaboration. They also appreciated the combination of theory and practice by making separate WAI and WAD models. Before enrolment in this study, they were unaware of the resilience they show daily. They said the current protocols do not assist in handling practice variations, because they only describe one way of performing each step of MR without acknowledging that daily practice is more complex than that.

I appreciated that the focus was not on adjusting how we work to match hospital protocols, but rather on how we can adapt our protocols to match daily practice.

We identified several factors that facilitated team reflection. The first one was team composition. We found that multidisciplinary teams consisting of nurses, physicians, pharmacy staff and quality advisors were able to review all components of the process and reflect on a more aggregated level. Team reflection was also high in hospitals with dedicated task forces for MR. These task forces consisted of several HCPs who had regular meetings to discuss and evaluate quality of care. They were very familiar with conducting a process analysis, as this was common practice for them. We noticed they were able to establish links between MR at discharge and other healthcare processes and place them in the hospital wide context. They were also able to formulate concrete improvement suggestions.

We have several task forces on this ward, one is related to quality and safety, and one is related to medication. And I mostly approached people from these task forces to participate in the study, because I knew that they would be motivated to join.

A second facilitator was process familiarity. CPs that were familiar with how MR was performed on their ward, found it easy to interpret the models. This helped them to then reflect on whether discrepancies between protocol and practice were unwanted or not.

We also identified some barriers. First, a lack of motivation negatively influenced the amount of team reflection. On some wards, urgency to analyse and improve this process lacked. Reasons included having to prioritize other work, and nurses saying that physicians should reflect on the process to propose improvements, because they said MR was mainly a physicians’ job. The second barrier was a lacking involvement from several CPs. Most CPs found it difficult to facilitate in this study and to be proactive. Reasons mentioned were having other priorities, not having experience in participating in a study and feeling disconnected from the study due to the scattered and online data collection because of the COVID-19 pandemic.

If you could have visited the ward to tell everybody about this study, then well, we would have planned a plenary meeting. But in this case, we were all less involved because we did not meet face to face.

## Discussion

In this study, we aimed to identify barriers and facilitators for team process improvement when applying FRAM. For each ward, team process improvement consisted of four phases: 1) Constructing FRAM, 2) Discussing differences between protocols and daily practice, 3) Reflecting on intended improvements, 4) Evaluating team reflection process. In line with other publications, FRAM proved to be a useful method for finding differences between protocol and practice, as well as identifying performance variability [5–7, 21]. During (online) feedback meetings, these discrepancies were shared with teams, prompting them to reflect on the desirability of these variations. Most of the proposed process improvements focused on enhancing collaboration among all healthcare professionals involved, underscoring the high level of interdependence in the medication reconciliation process at discharge. Improving collaboration was seen as essential for ensuring patient safety and reducing process variability [22, 23]. We found multidisciplinary teams and dedicated task forces to be facilitators for team process improvement, was well as teams being familiar with the process analysed. However, barriers such as lack of motivation and limited involvement were also identified.

The objective of this study was to identify barriers and facilitators for team process improvement when applying FRAM. Our results are in line with Wiedow and Konradt [24], confirming that team process improvement can be divided into two dimensions: team reflection and team adaptation (production of change). To the existing literature, this study adds knowledge about the relationship between those dimensions. Our results indicate that FRAM is a useful tool to gather input for teams to reflect on. Active team reflection then led to team adaptation. We identified team composition as our first facilitator for team process improvement. Team reflection was more common when dedicated task forces for MR were present, and reflection was limited within hospitals where nursing staff assembled into a non-recurring working group. These findings are in line with those of Schmutz et al. [25], and suggest that, in general, health care teams can be classified as action teams. Action teams mostly engage in activities that directly contribute to goal achievement (i.e. patient care). Transition phases, in which teams evaluate their quality of care, are often likely to suffer [25]. Multidisciplinary teams also seem to be better suited for team reflection than teams consisting of individuals from the same profession. Multidisciplinary team reflection allows team members to share their points of view, knowledge, and resources based on their own expertise, providing a holistic view of the healthcare process for all HCPs involved [26]. Our study confirmed these results. When nurses, physicians, pharmacy personnel and quality advisors were present during the feedback meeting and cooperated in team reflection, this led to a livelier discussion as they were able to approach the problems from different perspectives. Previous research identified a top-down approach as a barrier for team reflection, because it often led to resistance, discontent and refusal to collaborate [2]. In our study, CPs tried to overcome this barrier by inviting motivated HCPs to participate in team process improvement to ensure it became a team effort. On the other hand, we also found low motivation due to lacking urgency. The participants prioritized other work or did not think they were the ones who should be involved in the reflection process. This was an important barrier for team process improvement. Other studies also acknowledged the importance of emphasizing the benefits of change and establishing the need for change [27–30].

Lean process improvement is a similar approach regularly used for continuous process improvement in healthcare. Within the Lean approach, process improvement is achieved through standardised work, waste reduction (e.g. waste of time, movement or processing) and continuous flow [31, 32]. Continuous flow covers the idea that patients should flow between healthcare units and healthcare professionals without interruptions. To achieve continuous flow, it is fundamental to identify weak links or bottlenecks and find out root causes of problems in order to develop corrective actions in real time [32–34]. This line of reasoning corresponds with the traditional Safety-I perspective, which focuses on minimising the number of things that go wrong to prevent the same event from taking place in the future [35]. Paradoxically, the adjustments we make to try to stop errors from occurring often serves to increase system complexity, thereby making it more likely that something can go wrong [36, 37]. FRAM may thus be a promising alternative to Lean approach as this method is able to capture the complexity of the system and focus on resilience and adaptive strategies needed to ensure patient safety.

When choosing between applying FRAM or the Lean approach for healthcare improvement, it is essential to recognize the strengths and limitations of each method. Key factors negatively influencing the success of a Lean initiative include staff receptiveness, a lack of understanding of Lean principles, and an unclear focus [38–40]. Facilitators include tailoring the approach to the local context, learning from past experiences, promoting a long-term commitment to continuous improvement, and providing impactful training programs [41, 42]. While FRAM shows potential to be applied for team process improvement, it is not an easy to implement method. Especially when applying it to the health care system which is constantly exposed to pressures for quality, safety and productivity, while being subjected to resource scarcity [43]. As reported in other articles, incorporating FRAM models into feedback meetings with healthcare professionals can be challenging as most of them may be unfamiliar with the methodology [44]. This requires an extensive explanation from the researcher leading this session. Second, FRAM is a useful tool for visually capturing the complexity of the system. As a result, the graphical representations may be overwhelming if the model’s boundaries are not adequately set and the required level of detail is not kept in mind [7]. This indicates that the research team or someone who is trained in performing a FRAM analysis has a significant role in ensuring usability of FRAM as a tool for team process improvement. While there is limited literature discussing the barriers and facilitators specific to FRAM, we can reflect on our own experiences with the method. The challenges and facilitators identified for the Lean approach are largely applicable to FRAM as well.

Overall, methods for continuous improvement tend to share common success factors in the healthcare sector. In hospital environments, tensions often emerge between the demands for efficiency and performance targets, and the need to invest time and resources in cultivating a culture of continuous improvement [45]. Without acknowledging this, most quality improvement projects can fail to achieve intended aims. For healthcare organizations seeking to implement FRAM or similar methodologies, fostering a culture of quality improvement is essential. This goes hand in hand with the development of a learning organization [46]. This can be stimulated by organizing regular meetings led by participant leaders in order to facilitate the implementation of quality improvement initiatives [47, 48]. These interventions are most likely to succeed when the team has engaged leaders and engaged and empowered team members, and uses timely data to drive decision making. [46].

This study has several strengths. Firstly, we were able to include all HCPs professions involved in performing MR at discharge in this study. Since MR requires cooperation of multiple HCPs, their involvement during all research phases was instrumental for obtaining an accurate picture of their team reflection process. Second, our multicentre design allowed us to apply the FRAM as a tool for team process improvement in a variety of healthcare teams. This provided about many insights into how team process improvement can take place, including possible barriers and facilitators.

This study also has limitations. First, because this study was conducted during the COVID-19 pandemic, opportunity to actively participate in this study may have been lower than usual because of the increased workload. We may thus have obtained a lower willingness of HCPs to conduct team process improvement. We were also forced to conduct interviews and feedback sessions remotely. This meant we were unable to verify the input by conducting onsite observations. We were thus more dependent on the openness and knowledge of the interviewees. However, even though the COVID-19 pandemic has been a unique public health issue, workload in healthcare has been increasing over the past years, due to, among other things, personnel shortages, documentation activities and higher patient turnaround [49–51]. Follow-up studies could examine team reflection processes using FRAM intervention in more regular times. Additionally, we unexpectedly gained insights into the use of digital research methods. Conducting interviews and feedback meetings remotely turned out to be a satisfactory alternative to face-to-face meetings. It allowed us to include more HCPs in the study and to collect data more efficiently. A second limitation includes being unable to conduct follow-up measurements to monitor proposed improvements. Therefore, we cannot make any claims regarding their implementation in practice. Future research should focus on exploring the long-term impact of team process improvement when using FRAM. Research could focus on gaining insight into barriers and facilitators for actual implementation of improvement strategies. The effects of practice variation on patient outcomes could also be further explored. Lastly, it would be interesting to evaluate the scalability of FRAM in different healthcare contexts.

## Conclusion

This study showed that FRAM can provide valuable input to initiate team process improvement. This reflection process helped healthcare professionals to decide whether the adjustments they make are needed to keep patients safe or if they introduced risks which were meant to be reduced by implementing protocols. To undertake successful team reflection, we suggest assembling recurring and dedicated task forces consisting of healthcare professionals from different disciplines. Fostering a culture for continuous improvement is essential.

## Data Availability

Data are available from the authors upon reasonable request. Please contact Linda van Eikenhorst (L.vanEikenhorst@nivel.nl) if you want to request the data from this study.

## Abbreviations

ADE: Adverse drug event
CP: Contact person
EmpRes: Employee resilience scale
FRAM: Functional Resonance Analysis Method
HCP: Healthcare professional
MR: Medication reconciliation
WAD: Work as done
WAI: Work as imagined

## References

[1] Hollnagel E. Safety-I and safety-II: the past and future of safety management. CRC press; 2018.

[2] Plost G, Nelson DP. Empowering critical care nurses to improve compliance with protocols in the intensive care unit. Am J Crit Care. 2007;16(2):153–56. 10.4037/ajcc2007.16.2.153. 17322016

[3] Hollnagel E, Wears RL, Braithwaite J. From safety-I to safety-II: a white paper. The resilient health care net: published simultaneously by the University of Southern Denmark. 2015, University of Florida, USA, and Macquarie University, Australia.

[4] Sujan MA, Furniss D, Anderson J, Braithwaite J, Hollnagel E. Resilient health care as the basis for teaching patient safety – a safety-II critique of the World health Organisation patient safety curriculum. Saf Sci. 2019;118:15–21. 10.1016/j.ssci.2019.04.046.

[5] Kaya GK, Ovali HF, Ozturk F. Using the functional resonance analysis method on the drug administration process to assess performance variability. Saf Sci. 2019;118:835–40. 10.1016/j.ssci.2019.06.020.

[6] Hollnagel E, Hounsgaard J, Colligan L. FRAM-the functional resonance analysis method: a handbook for the practical use of the method. Centre for Quality, Region of Southern Denmark; 2014.

[7] Patriarca R, Di Gravio G, Woltjer R, Costantino F, Praetorius G, Ferreira P, et al. Framing the FRAM: a literature review on the functional resonance analysis method. Saf Sci. 2020;129:104827. 10.1016/j.ssci.2020.104827.

[8] Barnsteiner JH. Medication reconciliation. In: Hughes R, editor. Patient safety and quality: an evidence-based handbook for nurses. 3: agency for healthcare research and quality. Rockville, MD; 2008. 21328752

[9] Redmond P, Grimes TC, McDonnell R, Boland F, Hughes C, Fahey T. Impact of medication reconciliation for improving transitions of care. Cochrane Database Syst Rev. 2018;8(8). 10.1002/14651858.CD010791.pub2. PMC6513651 30136718

[10] Al-Hashar A, Al-Zakwani I, Eriksson T, Sarakbi A, Al-Zadjali B, Al Mubaihsi S, et al. Impact of medication reconciliation and review and counselling, on adverse drug events and healthcare resource use. Int J Clin Pharm. 2018;40(5):1154–64. 10.1007/s11096-018-0650-8. 29754251

[11] Mueller SK, Sponsler KC, Kripalani S, Schnipper JL. Hospital-based medication reconciliation practices: a systematic review. Arch Intern Med. 2012;172(14):1057–69. 10.1001/archinternmed.2012.2246. PMC3575731 22733210

[12] van Dijk LM, van Eikenhorst L, Wagner C. Daily practice performance (work-as-done) compared to guidelines (work-as-imagined) of medication reconciliation at discharge: outcomes of a FRAM study. Saf Sci. 2022;155:105871. 10.1016/j.ssci.2022.105871.

[13] Edmondson AC. The local and variegated nature of learning in organizations: a group-level perspective. Organ Sci (Linthicum). 2002;13(2):128–46. 10.1287/orsc.13.2.128.530.

[14] West M. Reflexivity and work group effectiveness: a conceptual integration. John Wiley & Sons, Ltd; 1996.

[15] van Dijk LM, Meulman MD, van Eikenhorst L, Merten H, Schutijser BC, Wagner C. Can using the functional resonance analysis method, as an intervention, improve patient safety in hospitals?: a stepped wedge design protocol. BMC Health Serv Res. 2021;21(1):1–10. 10.1186/s12913-021-07244-z. PMC8590349 34774048

[16] Näswall K, Kuntz J, Hodliffe M, Malinen S. Employee resilience scale (EmpRes) measurement properties. Resilient Organizations Res Programme: Christch, New Zealand. 2015.

[17] Näswall K, Kuntz J, Hodliffe M, Malinen S. Employee resilience scale (EmpRes). Technical report. 2013.

[18] Hill R. FRAM model visualizer. 0.4.3. 2016.

[19] VMS. Medicatieverificatie bij opname en ontslag [Medication reconciliation at admission and discharge] 2009. Available from: https://www.vmszorg.nl/wp-content/uploads/2017/03/web_2008.0101_praktijkgids_medverificatie.pdf.

[20] ActiZ AVK, Specialisten FM, Federatie van Nederlandse Trombosediensten GGN, Nederland GGZ, et al. Richtlijn Overdracht van Medicatiegegevens in de keten Herziening 2018/2019. 2019. Available from: https://www.zorginzicht.nl/binaries/content/assets/zorginzicht/kwaliteitsinstrumenten/Kwaliteitsstandaard+Overdracht+van+medicatiegegevens+in+de+keten.pdf.

[21] Schutijser BCFM, Jongerden IP, Klopotowska JE, Portegijs S, de Bruijne MC, Wagner C. Double checking injectable medication administration: does the protocol fit clinical practice? Saf Sci. 2019;118:853–60. 10.1016/j.ssci.2019.06.026.

[22] Clay BJ, Halasyamani L, Stucky ER, Greenwald JL, Williams MV. Results of a medication reconciliation survey from the 2006 society of hospital medicine national meeting. J Hosp Med: Off Publ Soc Hosp Med. 2008;3(6):465–72. 10.1002/jhm.370. 19084896

[23] Meguerditchian AN, Krotneva S, Reidel K, Huang A, Tamblyn R. Medication reconciliation at admission and discharge: a time and motion study. BMC Health Serv Res. 2013;13(1):1–11. 10.1186/1472-6963-13-485. PMC3842651 24261516

[24] Wiedow A, Konradt U. Two-dimensional structure of team process improvement: team reflection and team adaptation. Small Group Res. 2011;42(1):32–54. 10.1177/1046496410377358.

[25] Schmutz JB, Eppich WJ. Promoting learning and patient care through shared reflection: a conceptual framework for team reflexivity in health care. Acad Med. 2017;92(11):1555–63. 10.1097/ACM.0000000000001688. 28445215

[26] Miyazaki K, Taguchi T, Takemura Y. Effect of daily multidisciplinary team reflection in ambulatory care: a qualitative analysis. JMDH. 2022;15:323. 10.2147/JMDH.S348423. PMC8882021 35228803

[27] Fernandez S, Rainey HG. Managing successful organizational change in the public sector. Debating public administration. Routledge; 2017. p. 7–26.

[28] Dent EB, Goldberg SG. Challenging “resistance to change”. J Appl Behav Sci. 1999;35(1):25–41. 10.1177/0021886399351003.

[29] Armenakis AA, Harris SG. Reflections: our journey in organizational change research and practice. J Change Manag. 2009;9(2):127–42. 10.1080/14697010902879079.

[30] Oswald SL, Mossholder KW, Harris SG. Relations between strategic involvement and managers’ perceptions of environment and competitive strengths. Group Organ Manage. 1997;22(3):343–65. 10.1177/1059601197223003.

[31] Grout JR, Toussaint JS. Mistake-proofing healthcare: why stopping processes may be a good start. Bus Horiz. 2010;53(2):149–56. 10.1016/j.bushor.2009.10.007.

[32] Poksinska B. The current state of lean implementation in health care: literature review. Qual Manag Health Care. 2010;19(4):319–29. 10.1097/QMH.0b013e3181fa07bb. 20924253

[33] D’Andreamatteo A, Ianni L, Lega F, Sargiacomo M. Lean in healthcare: a comprehensive review. Health Policy (New York). 2015;119(9):1197–209. 10.1016/j.healthpol.2015.02.002. 25737260

[34] Bahensky JA, Roe J, Bolton R. Lean sigma--will it work for healthcare? J Healthc Inf Manag. 2005;19(1):39–44. 15682675

[35] Ellis LA, Churruca K, Clay-Williams R, Pomare C, Austin EE, Long JC, et al. Patterns of resilience: a scoping review and bibliometric analysis of resilient health care. Saf Sci. 2019;118:241–57. 10.1016/j.ssci.2019.04.044.

[36] Braithwaite J, Clay-Williams R, Hunte GS, Wears RL. Understanding resilient clinical practices in emergency department ecosystems. In: Resilient health care. Vol. 3. CRC Press; 2016. p. 115–28.

[37] Braithwaite J, Churruca K, Ellis LA, Long J, Clay-Williams R, Damen N, et al. Complexity science in healthcare. Sydney: Australian Institute of Health Innovation, Macquarie University; 2017.

[38] Morrow E, Robert G, Maben J, Griffiths P. Implementing large-scale quality improvement: lessons from the productive ward: releasing time to care™. Int J Health Care Qual Assur. 2012;25(4):237–53. 10.1108/09526861211221464. 22755479

[39] De Souza LB. Trends and approaches in lean healthcare. Leadersh Health Serv. 2009;22(2):121–39. 10.1108/17511870910953788.

[40] Grove AL, Meredith JO, Macintyre M, Angelis J, Neailey K. UK health visiting: challenges faced during lean implementation. Leadersh Health Serv. 2010;23(3):204–18. 10.1108/17511871011061037.

[41] Holden RJ. Lean thinking in emergency departments: a critical review. Ann Emerg Med. 2011;57(3):265–78. 10.1016/j.annemergmed.2010.08.001. PMC6548198 21035904

[42] Kovach J, De la Torre L, Walker D. Continuous improvement efforts in healthcare: a case study exploring the motivation, involvement and support necessary for success. Int J Six Sigma Compet Adv. 2008;4(3):254–69. 10.1504/IJSSCA.2008.021839.

[43] Rasmussen J. Risk management in a dynamic society: a modelling problem. Saf Sci. 1997;27(2–3):183–213. 10.1016/S0925-7535(97)00052-0.

[44] Bos V, Roorda D, De Sonnaville E, Van Boven M, Oosterlaan J, van Goudoever J, et al. Implementing structured follow-up of neonatal and paediatric patients: an evaluation of three university hospital case studies using the functional resonance analysis method. BMC Health Serv Res. 2022;22(1):191. 10.1186/s12913-022-07537-x. PMC8842913 35152890

[45] Burgess N, Radnor Z. Service improvement in the English National health service: complexities and tensions. J Manage Organ. 2012;18(5):594–607. 10.5172/jmo.2012.18.5.594.

[46] Flug JA, Stellmaker JA, Sharpe RE Jr, Jokerst CE, Tollefson CD, Bowman AW, et al. Kaizen process improvement in radiology: primer for creating a culture of continuous quality improvement. Radiographics. 2022;42(3):919–28. 10.1148/rg.210086. 35333633

[47] Hill JE, Stephani A-M, Sapple P, Clegg AJ. The effectiveness of continuous quality improvement for developing professional practice and improving health care outcomes: a systematic review. Implementation Sci. 2020;15(1):1–14. 10.1186/s13012-020-0975-2. PMC7168964 32306984

[48] Boonyasai RT, Windish DM, Chakraborti C, Feldman LS, Rubin HR, Bass EB. Effectiveness of teaching quality improvement to clinicians: a systematic review. JAMA. 2007;298(9):1023–37. 10.1001/jama.298.9.1023. 17785647

[49] Jourdain G, Chênevert D. Job demands–resources, burnout and intention to leave the nursing profession: a questionnaire survey. Int J Nurs Stud. 2010;47(6):709–22. 10.1016/j.ijnurstu.2009.11.007. 20138278

[50] Portoghese I, Galletta M, Coppola RC, Finco G, Campagna M. Burnout and workload among health care workers: the moderating role of job control. Saf Health Work. 2014;5(3):152–57. 10.1016/j.shaw.2014.05.004. PMC4213899 25379330

[51] De Groot K, De Veer AJ, Munster AM, Francke AL, Paans W. Nursing documentation and its relationship with perceived nursing workload: a mixed-methods study among community nurses. BMC Nurs. 2022;21(1):1–12. 10.1186/s12912-022-00811-7. PMC8795724 35090442

